# Characterization of UDP-glucose pyrophosphorylases from different organisms

**DOI:** 10.1042/BSR-2024-1494

**Published:** 2025-04-11

**Authors:** Siqi Zhang, Xin Song, Yuqi Qin

**Affiliations:** 1 National Glycoengineering Research Center, Shandong University, Qingdao, China; 2 State Key Laboratory of Microbial Technology, Shandong University, Qingdao, China

**Keywords:** cassava, enzymes, plant, UDP-glucose, UDP-glucose pyrophosphorylases, UTP-glucose-1-phosphate uridylyltransferases

## Abstract

UDP-glucose pyrophosphorylases (UGPases) catalyze the conversion of UTP and glucose-1-phosphate (Glc1P) to UDP-glucose and pyrophosphate, playing crucial roles in cell metabolism. The UGPases are related to the biosynthesis of glycans in various organisms and linked to bacterial survival, plant programmed cell death, and even human cancers. Eleven UGPases from the bacterium *Escherichia coli*; fungi *Saccharomyces cerevisiae* (ScUGP) and *Aspergillus niger* (AnUGP); plants *Hordeum vulgare* (barley) (HvUGP), *Arabidopsis thaliana* (AtUGP), *Solanum tuberosum* (potato) (StUGP), *Manihot esculenta* (cassava) (MeUGP), *Ipomoea batatas* (sweet potato) (IbUGP), and *Zea mays* (maize) (ZmUGP); and animals *Drosophila melanogaster* (fruit fly) (DmUGP) and *Homo sapiens* (human) (HsUGP) were expressed in *E. coli* and assayed. MeUGP and StUGP have the highest and second-highest specific activities, respectively. The second-order rate constant *k*_cat_/*K*_m_ values of 11 UGPases are ranked from high to low in the following order: MeUGP > StUGP > ZmUGP > IbUGP > AtUGP > AnUGP > HvUGP > HsUGP > DmUGP > ScUGP > EcUGP. EcUGP, ScUGP, AnUGP, HvUGP, AtUGP, DmUGP, and HsUGP show a temperature optimum of 37℃. MeUGP, IbUGP, and ZmUGP showed a temperature optimum of 50℃. Overall, recombinant UGPases were not thermally stable. Ten UGPases were rapidly inactivated at 60℃ except for IbUGP. The recombinant UGPases use Glc1P with high activities. UGPases exhibit variations in nucleoside triphosphate (NTP) utilization efficiency. The results improve the knowledge of the characteristics of UGPase from various organisms and provide the potential to use MeUGP or StUGP as the engineering target of cell factories.

## Introduction

Nucleotide sugars play essential roles in carbohydrate metabolism, serving as glycosyl donors for the biosynthesis of polysaccharides, glycoproteins, and glycoconjugates. Among nucleotide sugars, UDP-sugars stand out—not only for the significance of the functions they are involved in the biosynthesis of glycans but also for their abundance (UDP-sugars make up as much as 55% of the total nucleotide pools) and the large number of reactions in which they are used as substrates [1,2]. For example, UDP-glucose (UDP-Glc) alone is involved in 270 reactions [3], acting as an essential precursor for the biosynthesis of glycans, e.g. sucrose, cellulose, hemicellulose, and glycogen, and glycan parts of glycoproteins, glycolipids, and proteoglycans in different organisms. Another role for UDP-Glc is to serve as a substrate for glycosylation of many secondary metabolites such as steroids, flavonoids, phenylpropanoids, betalains, terpenoids, and glucosinolates. In addition, UDP-Glc acts as a substrate for the glycosylation of numerous hormones (e.g. steroids, flavonoids, and phenylpropanoids) or glycosyl-hormone conjugates (e.g. auxin, cytokinin, and salicylic acid) [2].

UDP-glucose pyrophosphorylase (UGPase), also known as UTP-glucose-1-phosphate uridylyltransferase (EC 2.7.7.9), catalyzes a reversible production of UDP-Glc and pyrophosphate (PPi) from glucose-1-phosphate (Glc1P) and UTP. UGPase is a member of the NTP:sugar nucleotidyltransferases superfamily, a related group of proteins with a similar folding pattern and some conserved sequence elements [4]. The UGPases have essential activity for cell metabolism. The UGPases are not only related to the biosynthesis of glycans in microorganisms, plants, and animals but also linked to microorganism survival, plant programmed cell death, and even human cancers [5–7].

Despite the ubiquitous distribution of UGPase activity throughout all domains of life, prokaryotic UGPases are evolutionarily unrelated to their eukaryotic counterparts. The similarities between UGPases of prokaryotic origin and their eukaryotic counterparts are only ~8%, which is considered non-significant [4,8]. However, UGPases are very well conserved within the prokaryotic or eukaryotic kingdom. For instance, *Escherichia coli* UGPase (GalU) bears a 43.4% identity with UGPase (YngB) from the *Bacillus subtilis*, and the human UGPase (UGP2) and *Arabidopsis thaliana* UGPase share a 55.0% identity. Prokaryotic and eukaryotic UGPases differ significantly in protein size and tertiary and quaternary structure. The molecular mass of the prokaryotic UGPase monomer is approximately 30–35 kDa and crystallized as tetramers [4]. *E. coli* GalU (32.9 kDa) forms a tetramer with 222-point group symmetry; each monomer is dominated by an eight-stranded mixed β-sheet [9]. Molecular masses of eukaryotic UGPases are higher than those of prokaryotic UGPase. Molecular masses of plant UGPases protein are usually 50–55 kDa, depending on plant species [2,10,11]. The budding yeast *Saccharomyces cerevisiae* UGPase (Ugp1) (56.0 kDa) forms an octamer as the enzymatically active form of the protein [12]. Plants often contain UGPase isozymes. For example, there are two UGPase isozymes from *A. thaliana*, UGP1 and UGP2; the specific activity of UGP2 is higher than that of UGP1 [13].

The catalytic activity of UGPases from different organisms has been studied, such as prokaryotic UGPases from *E. coli* [14], *B. subtilis* [15], *Xanthomonas citri* [16], and *Sulfurisphaera tokodaii* [17] and eukaryotic UGPases from *S. cerevisiae* [18], *A. thaliana* [19], *Hordeum vulgare* [20], *Solanum tuberosum* [21,22], *Leishmania major* [23], and human [24]. However, it is not easy to compare the activity of UGPases from different organisms due to variations in assay methods. The activity of *A. thaliana* UGPase was determined by monitoring UDP-Glc produced by coupled enzymatic assays (UDP-Glc dehydrogenase was added) [19], and the activity of *E. coli* UGPase was determined by monitoring the amount of Glc1P with Mg^2+^-dependent phosphoglucomutase and glucose 6-phosphate dehydrogenase added [14]. In recent years, researchers have begun quantifying the Pi released from inorganic PPi using the colorimetric method to determine the activities of UGPases from *B. subtilis*, *Brachypodium distachyon*, *Euglena gracilis*, and humans [15,24–26]. However, these variations in assay methods have led to variations in measured activity value, even for the UGPase from the same species. For instance, the measured specific activity difference for the potato UGPase is greater than four times recorded by different research teams; the Michaelis constant *K*_m_ toward the same substrate UTP varies from 0.22 mM to 0.53 mM [27,28].

In this study, 11 UGPases from various organisms, including bacteria, fungi, plants, and animals, were expressed in *E. coli*. The recombinant UGPases were purified, and their specific activities were determined and compared. Four previously unstudied UGPases that are from *Aspergillus niger*, *cassava*, sweet potato, and maize were characterized. It has been discovered that MeUGP has the highest recorded specific activity to date.

## Materials and methods

### Strains and plasmids construction

The encoding sequences of UGPase from *E. coli*, *S. cerevisiae*, *A. niger, A. thaliana*, *H. vulgare* (barley), *S. tuberosum* (potato), *Manihot esculenta* (cassava), *Ipomoea batatas* (sweet potato), *Zea mays* (maize), *Drosophila melanogaster* (fruit fly), and *Homo sapiens* (human) were synthesized by GenScript Biotech Corporation (Nanjing, China) after codon optimization for heterologous expression in *E. coli*. The UniProt Entry of each UGPase can be found in Table 1. The sequences were digested with restriction endonucleases *Sal*I and *Xho*I and ligated with plasmid pET28b(+) cut with the same restriction enzymes. For protein expression, the resulting recombinant plasmids were transformed into *E. coli* BL21 (DE3).

**Table 1:** The sources and nomenclatures of UGPases in this study.

Classification	Organisms		UGPases			
	Scientific names	Common names	UniProt entry/names	Molecular masses (kDa)^1^	References	Nomenclature in this study
Bacteria	*Escherichia coli*	-	P0AEP3/GalU	32.9	[14]	EcUGP
Fungi	*Saccharomyces cerevisiae*	-	P32861/UGP1	55.9	[10]	ScUGP
	*Aspergillus niger*	-	A2QYD2	57.9	N	AnUGP
Plants	*Hordeum vulgare*	Barley	Q43772	51.6	[29]	HvUGP
	*Arabidopsis thaliana*	-	Q9M9P3/UGP2	51.9	[30]	AtUGP
	*Solanum tuberosum*	Potato	P19595	51.8	[27]	StUGP
	*Manihot esculenta*	Cassava	A0A251LGJ0	51.5	N	MeUGP
	*Ipomoea batatas*	Sweet potato	G8XR42	51.3	N	IbUGP
	*Zea mays*	Maize	B4F8W6	52.1	N	ZmUGP
Animals	*Drosophila melanogaster*	Fruit fly	A5XCL5	57.8	[31]	DmUGP
	*Homo sapiens*	Human	Q16851/UGP2	56.9	[31]	HsUGP

1 The data from UniProt database (https://www.uniprot.org/).

N, No reports.

### Protein expression and purification

The fresh recombinant *E. coli* BL21 (DE3) cells were inoculated in 50-ml LB containing 30 mg/ml kanamycin in a 250-ml Erlenmeyer flask. The cells were incubated with shaking at 37°C until OD_600_ reached 0.6–0.8. Protein expression was induced with a final concentration of 0.4 mM isopropyl-β-D-1-thiogalactopyranoside (IPTG) at 18°C for 16 h. After that, cells were harvested by centrifugation at 14000×g, 4°C for 15 min, and pellets were washed once with phosphate-buffered saline and resuspended in 5 ml of lysis buffer (50 mM Tris, 500 mM NaCl, pH7.0). The lysis buffer was added with RNase A to a final concentration of 100 µg/ml and protease inhibitor (Protease Inhibitor Cocktail I, 100× in DMSO, TargetMo). The cells were lysed by sonification with a microtip on ice. The cell lysate was centrifuged at 14000×g, 4°C for 15 min. The supernatant was collected and filtered through 0.22-µm filters (Merck Millipore). Clarified supernatant was loaded onto a 1-ml HisTra^™^ HP column (Smart-Lifesciences, Changzhou, China) previously equilibrated with buffer A (50 mM Tris; 500 mM NaCl, pH 7.0). Then, the column was washed with 5 ml of buffer A, followed by buffer A containing 500 mM imidazole. The HisTrap flow-through was dialyzed in the buffer (50 mM Tris 150 mM NaCl, pH 7.0). Protein content was determined spectrophotometrically at 595 nm using the Modified Bradford Protein Assay Kit (Sango Biotech, Shanghai, China), with bovine serum albumin (BSA) as standard.

### UGPase activity assay

The activity of UGPase was determined using an assay based on quantification of the Pi released from inorganic PPi, the product of the pyrophosphorylase reaction according to the method of Wu et al. [15,25]. The reaction mixture contained 50 mM MOPS-KOH buffer, pH 7.0, 10 mM MgCl_2_, 0.2 mg/ml BSA, 0.5 mM Glc1P, 1 mM UTP, 0.5 mU/ml of yeast inorganic pyrophosphatase, and a proper enzyme dilution. Assays were initiated by adding 0.5 mM Glc1P, incubated for 5 min at 37°C, and terminated by adding a Malachite Green Phosphate Detection Kit (Beyotime Biotechnology, Shanghai, China). The absorbance at 630 nm was measured with a Microplate Spectrophotometer (Agilent BioTek, Beijing, China) to determine the amount of the blue-colored phosphate–molybdenum complex proportional to the phosphate present. The amount of produced Pi was quantified with a Pi standard curve. One unit (U) of enzyme activity was defined as the amount of enzyme that produced 1 µmol of PPi per minute under the above reaction conditions.

### Effect of temperature and pH on UGPases

The optimal temperature for UGPase activity was determined in different assay temperatures, ranging from 20℃ to 80℃ for 5 min. The optimal pH for UGPase activity was determined according to the method described by Decker et al. [32]. Prior to assays of the pH optimum, the buffer (12.5 mM acetate, 12.5 mM Hepes, 12.5 mM Mes, 12.5 mM Tricine, 10 mM MgCl_2_, 0.2 mM Glc1P, 0.2 mM UTP, and an aliquot of purified UGPase) was prepared and adjusted to the required pH with 1 M NaOH or 1 M HCl and then was incubated for 5 min at 37°C. The assay procedures were as described in the ‘UGPase activity assay’ section. The thermostability of recombinant UGPase was determined by incubation with UGPases at 37°C, 50°C, 60°C, and 70°C. Purified enzymes of UGPase were diluted in 50 mM MOPS-KOH sodium acetate buffer (pH 7) and incubated at 37°C, 50°C, 60°C, and 70°C for various lengths of time. After heat treatment, the UGPases were tested as in the ‘UGPase activity assay’ section. The initial activity (unheated) was regarded as 100% activity. Residual activity was determined as a proportion of the initial activity.

### Determination of kinetic constants and substrate specificity

kinetic constants were determined by measuring enzyme activity at different concentrations of one substrate while keeping a fixed and saturating amount of the other. for measuring the *K*_m_ value against the substrate glc1p, the reactions contained 0.2 mm utp and glc1p at a concentration of 2 mm, 1 mm, 0.5 mm, 0.2 mm, 0.1 mm, 0.05 mm, and 0.02 mm. the kinetic constants were calculated using the fitting software (graphpad prism). substrate specificity was determined by measuring enzyme activity toward nucleotide triphosphates and sugar phosphates. utp, atp, gtp, and ctp were used to investigate the specificity of ugpases toward nucleotide triphosphates. a quantity of 0.2 mm of utp, atp, gtp, or ctp was added to the mixtures containing 0.2 mm glc1p. glc1p, galactose 1-phosphate (gal1p), glucuronic acid 1-phosphate (glca1p), and n-acetyl-glucosamine 1-phosphate (glcnac1p) were used to investigate the specificity of ugpases toward sugar phosphates. a quantity of 0.2 mm of glc1p, gal1p, glca1p, or glcnac1p was added to the mixtures containing 0.2 mm utp. ppi generation indirectly indicates whether and to what extent ugpase binds to distinct substrates.

### Phylogenetic analysis

The protein sequences of various UGPases were downloaded from the UniProt database (https://www.uniprot.org/). Sequences were analyzed and aligned in the ClustalW server (http://www.genome.jp/tools/clustalw/). The phylogenetic tree was constructed using Molecular Evolutionary Genetic Analysis (MEGA 7.0) [33] with the Neighbor-Joining method, with a bootstrap of 1000.

## Results

### The selection of UGPases from different organisms

**Figure 1:**
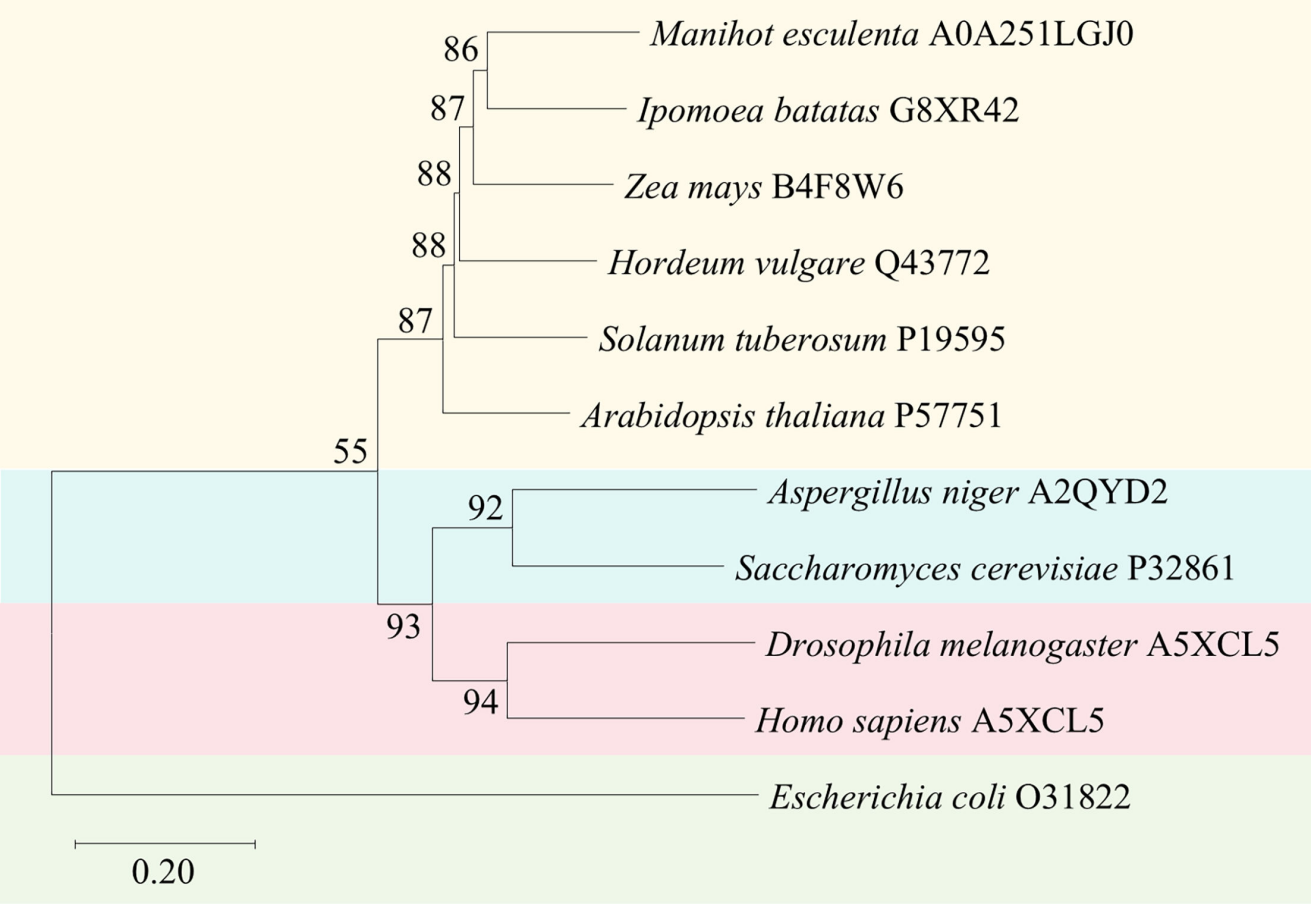
Phylogenetic analysis of UDP-glucose pyrophosphorylases (UGPases) from different organisms. Yellow background, UGPases from plants. Blue background, UGPases from fungi. Red background, UGPases from animals. Green background, UGPases from bacteria *E. coli*. Multiple sequence alignment was performed, and a phylogenetic tree was constructed using the maximum likelihood method by MEGA 7.0. Coefficients were indicated below the respective nodes, and gaps in the alignment were not considered.

The sources and nomenclatures of UGPase in this study are listed in Table 1. Eleven UGPases from different organisms were selected, including one UGPase from the bacteria *E. coli* (EcUGP); two UGPases from fungi *S. cerevisiae* (ScUGP) and *A. niger* (AnUPG); six UGPases from plants *H. vulgare* (barley) (HvUGP), *A. thaliana* (AtUGP), *S. tuberosum* (potato) (StUGP), *M. esculenta* (cassava) (MeUGP), *I. batatas* (sweet potato) (IbUGP), and *Z. mays* (maize) (ZmUGP); and two UGPases from animals *D. melanogaster* (fruit fly) (DmUGP) and *H. sapiens* (human) (HsUGP). Phylogenetic analysis shows that fungal UGPases and mammalian UGPases are more closely related to each other phylogenetically than to plant UGPases. There is no phylogenetic relationship between EcUGPase and UGPases from eukaryotic species (Figure 1), which is consistent with previous reports that prokaryotic UGPs are evolutionarily unrelated to their eukaryotic counterparts [4,8].

Among 11 UGPases, 7 UGPases (EcUGP, ScUGP, HvUGP, AtUGP, StUGP, DmUGP, and HsUGP) have been characterized previously (see the references in Table 1). These seven UGPases were chosen for two reasons: (1) The organisms that produce these seven UGPases cover kingdoms of bacteria, fungi, plants, and animals. *E. coli*, *S. cerevisiae*, *A. thaliana*, and *D. melanogaster* are model organisms; (2) UGPases from barley and potato have been the most extensively studied representatives of the plant UDP-sugar-producing family [20,32,34]. Four UGPases (AnUGP, MeUGP, IbUGP, and ZmUGP) have not yet been the subject of any research reports. Among them, one (AnUGP) from the fungus and three (MeUGP, IbUGP, and ZmUGP) from plants were chosen for two reasons: (1) Fungus *A. niger* is considered generally recognized as safe and is one of the most important microorganisms used in biotechnology to produce protein, food enzymes, organic acids, and secondary metabolites [35,36]. UGPase may become the engineering target in the modification of *A. niger* as chassis by metabolic engineering; (2) ADP-GPase is plastidic starch phosphorylase, which supplies ADP-glucose as the glucosyl donor to elongate alpha-1,4-glucosidic chains in plant starch synthesis. Interestingly, there is a high level of specific activity of plant UGPases, such as UGPase from barley and potato, as indicated by the data of the BRENDA database (https://www.brenda-enzymes.org/index.php). Therefore, special attention was given to the plant UGPases, especially plants with higher starch content (cassava, sweet potato, and maize).

### UGPases from cassava and *A. niger* have the highest and lowest activities, respectively

**Figure 2:**
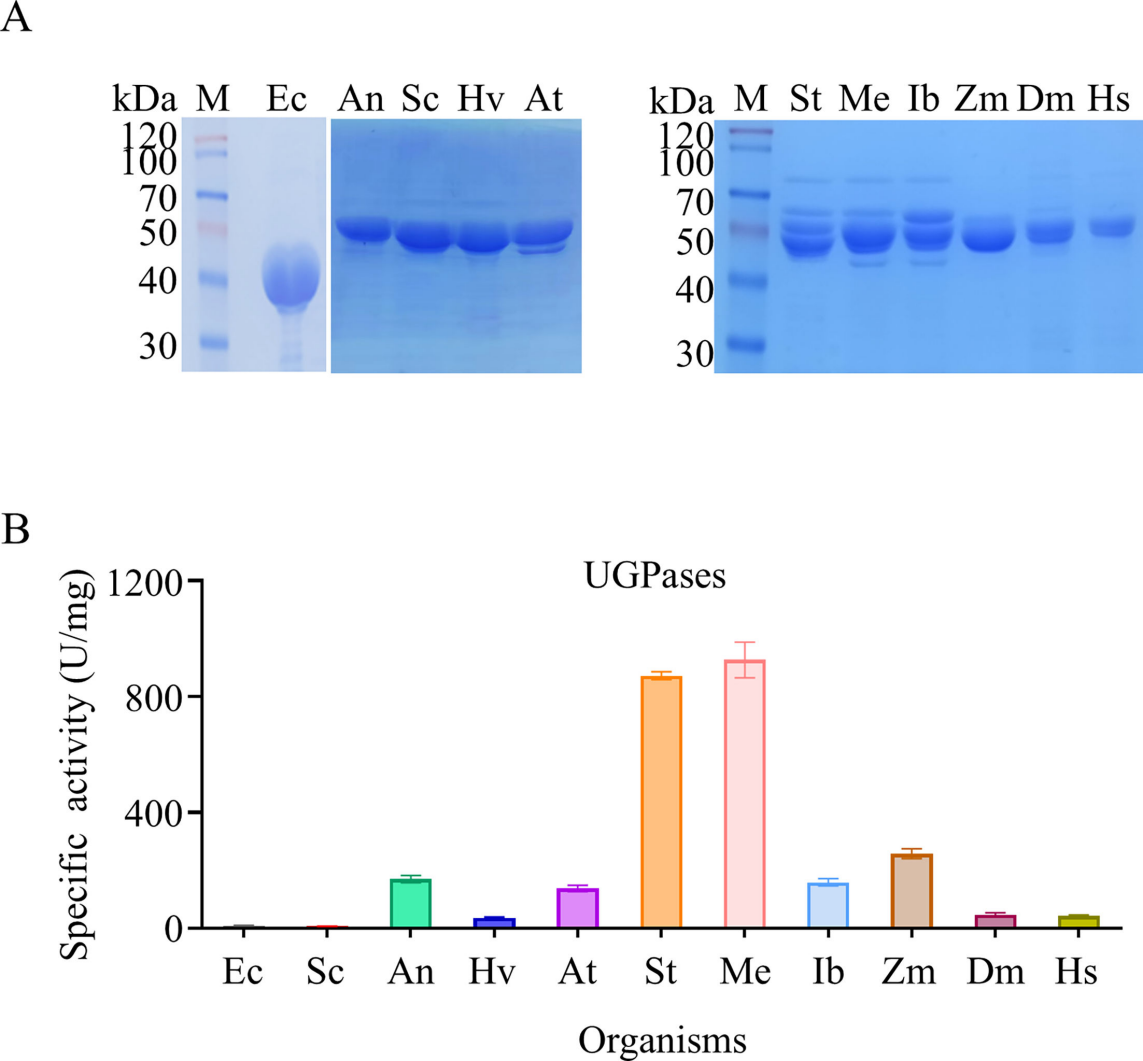
The purified UGPases and specific activity of UGPase from different organisms. (**A**) SDS-PAGE (12% gel) analysis of the purified UGPases from different organisms. M, protein marker; marker ladders are in kDa. (**B**) The specific activity of UGPases. The biological triplicates were performed for UGPase activity analyses. An, *A. niger*; At, *A. thaliana*; Ec, *E. coli*; Dm, *D. melanogaster* (fruit fly); Hs, *H. sapiens* (human); Hv, *H. vulgare* (barley); Ib, *I. batatas* (sweet potato); Me, *M. esculenta* (cassava); Sc, *S. cerevisiae*; St, *S. tuberosum* (potato); Zm, Z. mays (maize).

Eleven UGPases from different organisms were expressed using *E. coli* BL21 (DE3) as the expression host and then were purified. The purified UGPases were examined using SDS-PAGE (Figure 2A). Approximately 20–45 µg of purified various UGPases was loaded on the gels. The molecular weights of 11 UGPases presented in SDS-PAGE are consistent with their predicted molecular weights in Table 1. The molecular mass of prokaryotic EcUGP is smaller than that of eukaryotic UGPases. The specific activities of 11 UGPases were determined (Figure 2B). Cassava MeUGP and potato StUGP show the highest and second-highest specific activity of 927.1 U/mg and 828.5 U/mg, respectively. EcUGP and ScUGP show the lowest and second-lowest specific activities of 8.2 U/mg and 10.7 U/mg, respectively. Sweet potato IbUGP and maize ZmUGP show specific activities of 159.6 U/mg and 258.2 U/mg, respectively. Cassavas and potatoes are high in starch. Fresh cassavas and potatoes contain ~20% dry matter, of which 60–80% is starch [37,38]. Two UGPases from highly starch-producing plants have the highest specific activity, while the two fungal UGPases have the lowest.

### The temperature and pH optima of recombinant UGPases

**Figure 3:**
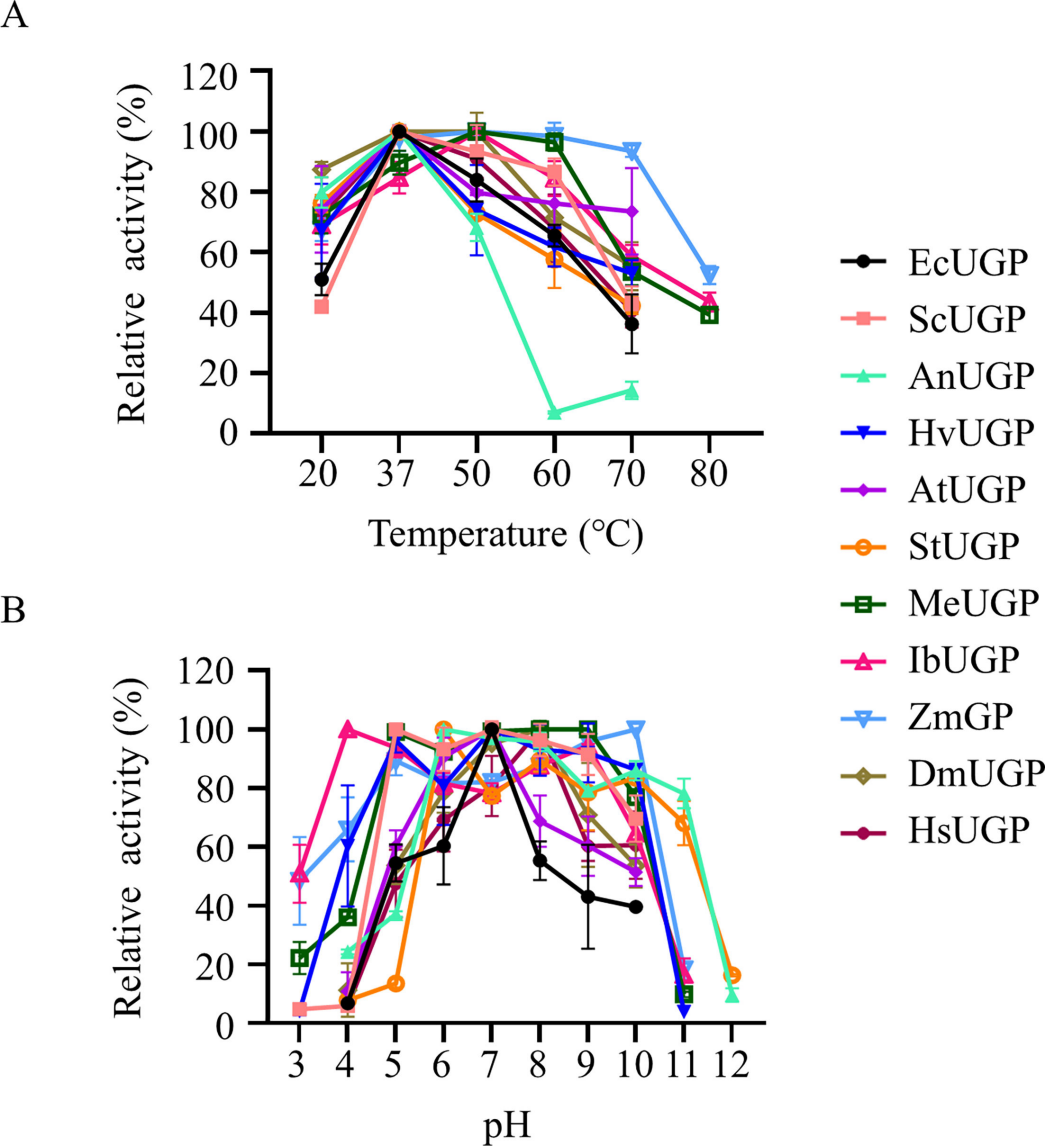
Temperature and pH optima of various UDP-glucose pyrophosphorylases (UGPases). (**A**) Temperature optima. The highest activity for each UGPase at a specific temperature is defined as 100%. (**B**) pH optima. The highest activity for each UGPase at a specific pH is defined as 100%. The biological triplicates were performed for UGPase activity analyses.

The temperature and pH optima of 11 recombinant UGPases were assayed (Figure 3A). UGPase activity was monitored in different assay temperatures, ranging from 20°C to 80°C. EcUGP, ScUGP, AnUGP, HvUGP, AtUGP, DmUGP, and HsUGP show a temperature optimum of 37°C. MeUGP, IbUGP, and ZmUGP showed a temperature optimum of 50°C. All three UGPases showing higher optimal temperatures are UGPases from plants (Figure 3A). Eleven UGPase activity was monitored in different assay pHs (Figure 3B). Most UGPases had a relatively broad pH optimum between pH 5.0 and 10 and had a sharp drop in activity above pH11 and below pH4 (Figure 3B). For example, StUGP, MeUGP, IBUGP, and ZmUGP retained over 80% activity at pH4–10; ScUGP, DmUGP, and HsUGP retained over 60% activity at pH5–10. The only exception is EcUGP, which had the highest activity at pH7 but rapidly loses activity as pH rises or falls (Figure 3B). The earlier study on barley UGPase also gave a relatively broad optimal pH (5.5–9.5) [32]. The results indicate that plant UGPases have a broad pH adaptation even though plant cell cytosol has a relatively narrow pH range of 6.5–7.3 [39].

### Thermal stability of recombinant UGPases

**Figure 4:**
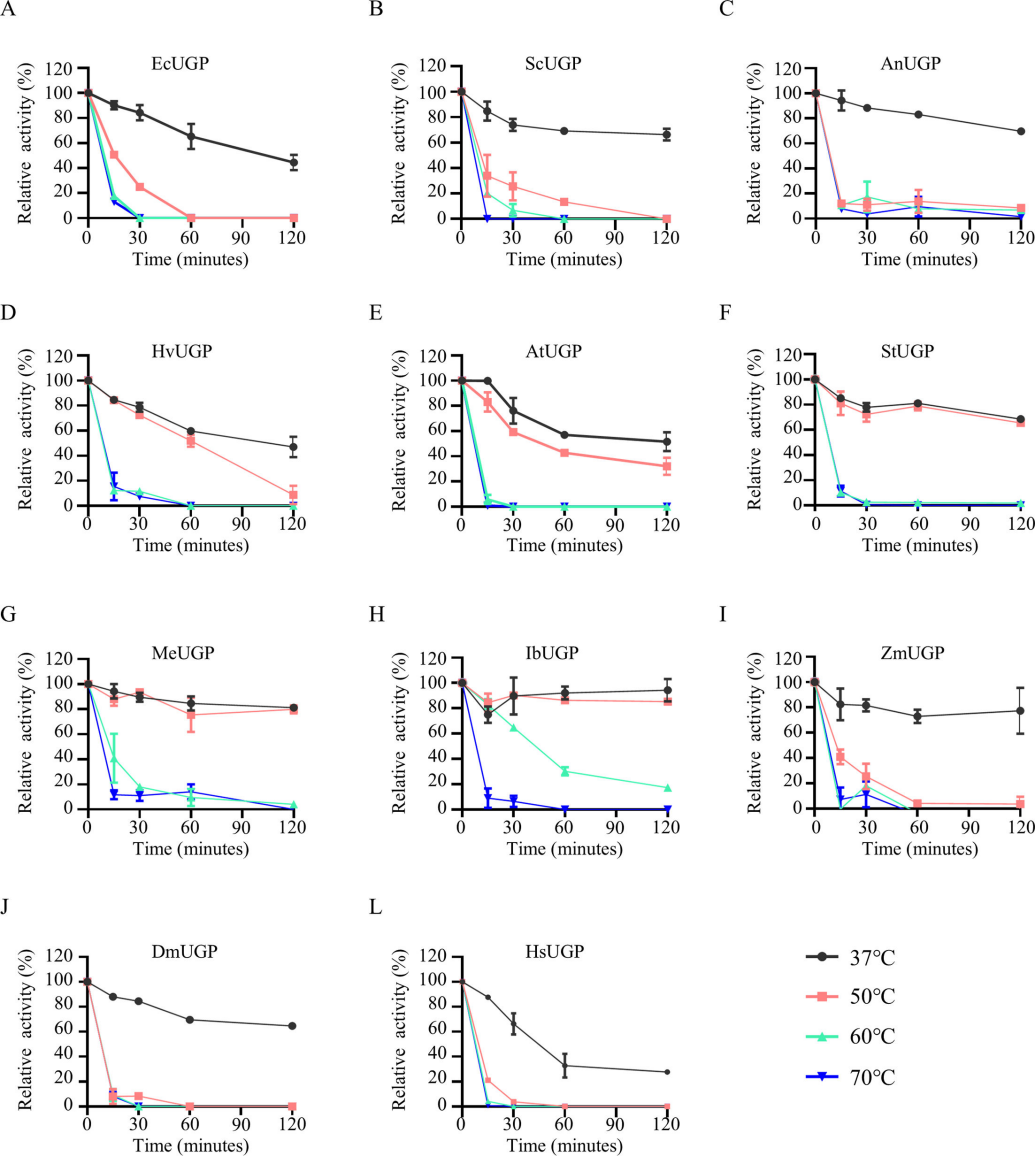
Thermal stability of recombinant UGPases. (**A**) EcUGP; (**B**) ScUGP; (**C**) AnUGP; (**D**) HvUGP; (**E**) AtUGP; (**F**) StUGP; (**G**) MeUGP; (**H**) IbUGP; (**I**) ZmUGP; (**J**) DmUGP; (**K**) HsUGP. The recombinant UGPases were incubated in the MOPS-KOH buffer (50 mM, pH 7.0) at 37°C, 50°C, 60°C, and 70°C, respectively, and sampled at 15, 30, 60, and 120 min. The enzyme activity of each UGPase before thermal incubation is defined as 100%. The values are means from measurements of biological triplicates. An, *A. niger*; At, *A. thaliana*; Ec, *E. coli*; Dm, *D. melanogaster* (fruit fly); Hs, *H. sapiens* (human); Hv, *H. vulgare* (barley); Ib, *I. batatas* (sweet potato); Me, *M. esculenta* (cassava); Sc, *S. cerevisiae*; St, *S. tuberosum* (potato); Zm, Z. mays (maize).

The thermal stability of UGPases was monitored in different assay temperatures: 37°C, 50°C, 60°C, and 70°C at pH7.0. Overall, recombinant UGPases were not thermally stable. Only IbUGP retained over 60% of its activity at 60°C for 30 min; the other ten UGPases were rapidly inactivated at 60°C. StUGP, MeUGP, and IbUGP retained most activities at 50°C for 2 h, whereas EcUGP, ScUGP, AnUGP, DmUGP, and HsUGP failed to retain more than 30% activity at 50°C for 30 min. In particular, EcUGP and HsUGP showed a loss of enzyme activity after 2 h at 37°C (Figure 4).

### Substrate specificity and kinetic analysis

**Figure 5:**
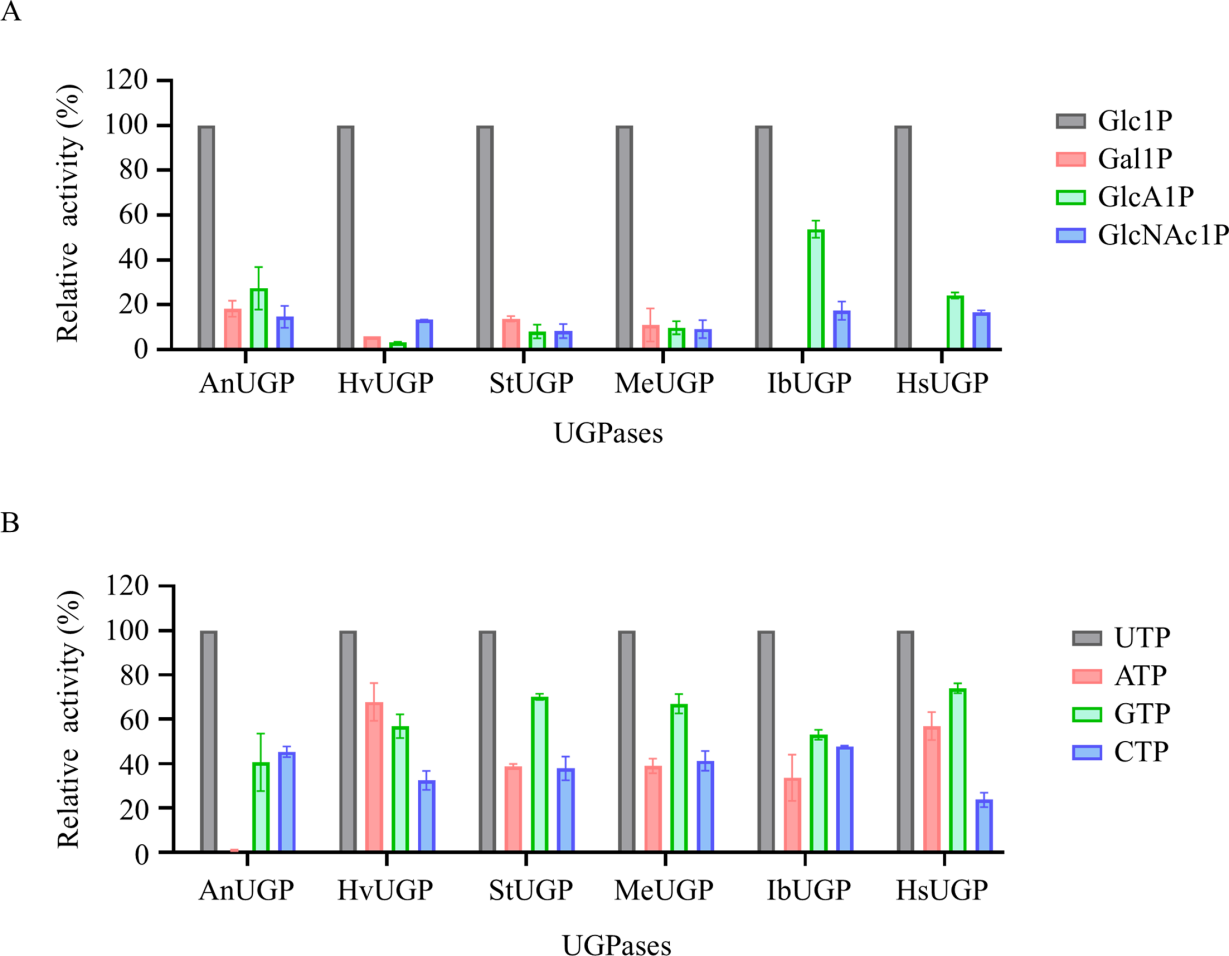
The substrate preferences of recombinant UDP-glucose pyrophosphorylases (UGPases). (**A**) The preference of sugar phosphates Glc1P, Gla1P, GlcA1P, and GlcNAc1P. The highest UGPase activity was measured when using Glc1P as a substrate, which is defined as 100%. (**B**) The preference of nucleotide triphosphates UTP, CTP, ATP, and GTP. The highest UGPase activity was measured when using UTP as a substrate, which is defined as 100%. The values are means from measurements of biological triplicates.

The substrate preferences of six recombinant UGPases for different sugar phosphates and nucleotide triphosphates were compared. Six recombinant UGPases were specific for Glc1P. For example, the activities of MeUGP and StUGP in the presence of other sugar phosphates (Gla1P, GlcA1P, or GlcNAc1P) were less than 10% of those measured in the presence of Glc1P (Figure 5A). The results are consistent with that reported in the plants *A. thaliana* UGPase and barley UGPase [40]. MeUGP and StUGP used UTP with high activity levels. MeUGP and StUGP can also use other nucleotide triphosphates (CTP, ATP, or GTP) (Figure 5B). AnUGP also used UTP with high activity levels but could not use ATP. Eukaryotic UGPases exhibit wide variations in NTP utilization efficiency. *A. thaliana* UGP uses UTP as the only effective nucleotide donor, while ScUGP and bovine UGPase can also use ATP as donor substrate, with the relative activities 26–36% of UTP; HvUGP also uses ATP, CTP, and GTP at low relative activities [40,41]. The preference for various substrates should be ascribed to the different structures of various UGPases.

**Figure 6:**
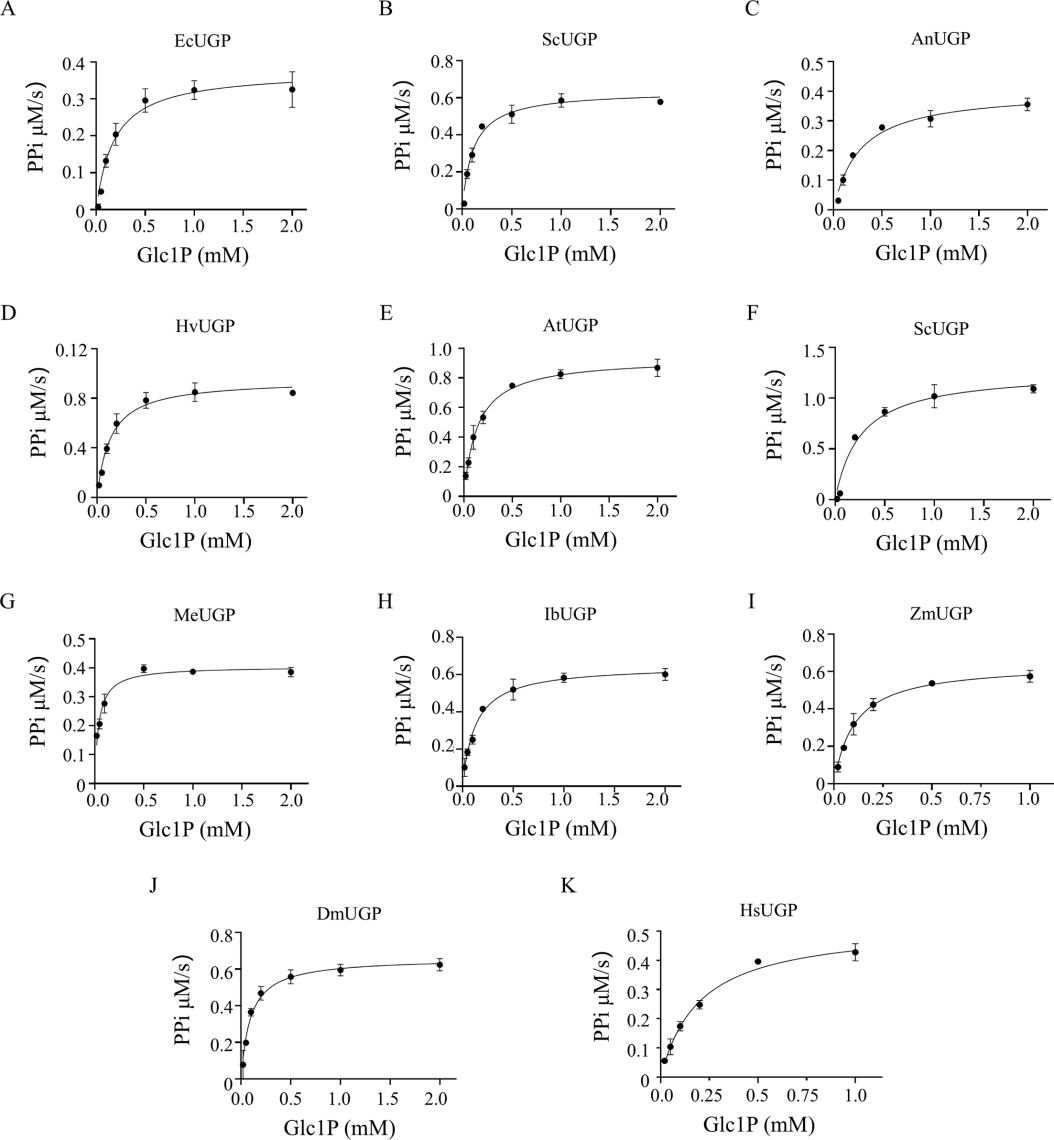
The kinetic curves of recombinant UGPases. The kinetic curves of recombinant UGPases toward substrate Glc1P. (**A**) EcUGP; (**B**) ScUGP; (**C**) AnUGP; (**D**) HvUGP; (**E**) AtUGP; (**F**) StUGP; (**G**) MeUGP; (**H**) IbUGP; (**I**) ZmUGP; (**J**) DmUGP; (**K**) HsUGP. An, *A. niger*; At, *A. thaliana*; Ec, *E. coli*; Dm, *D. melanogaster* (fruit fly); Hs, *H. sapiens* (human); Hv, *H. vulgare* (barley); Ib, *I. batatas* (sweet potato); Me, *M. esculenta* (cassava); Sc, *S. cerevisiae*; St, *S. tuberosum* (potato); UGPases, UDP-glucose pyrophosphorylases; Zm, Z. mays (maize).

The Michaelis–Menten kinetic parameters *k*_cat_ and *K*_m_ of 11 recombinant UGPases were determined using different concentrations of Glc1P as the substrate (Table 2). The kinetic curves are shown in Figure 6. The top five second-order rate constant *k*_cat_/K_m_ values were observed in UGPases from plants (MeUGP, StUGP, ZmUGP, IbUGP, and AtUGP). MeUGP showed the highest *k*_cat_/*K* value, about 660-fold and 257-fold higher than the lowest EcUGP and the second-lowest ScUGP. StUGP showed the second-highest *k*_cat_/*K* value, about 181-fold and 70-fold higher than the lowest EcUGP and second-lowest ScUGP. Compared with several UGPases from plants, the *k*_cat_/*K* values of two UGPases from animals (DmUGP and HsUGP) are also relatively low. StUGPand MeUGP have the highest and second-highest *k*_cat_ values, whereas EcUGP and ScUGP have the lowest and second-lowest *k*_cat_ values (Table 2).

**Table 2:** Kinetic parameters of the recombinant UGPases.

UGPases	*k*_cat_ (s^−1^)	*K*_m_ (mM)	*k*_cat_/*K*_m_ (mM^−1^s^−1^)
EcUGP	0.0162 ± 0.004	0.201 ± 0.080	0.081
ScUGP	0.0237 ± 0.003	0.114 ± 0.028	0.208
AnUGP	0.435 ± 0.091	0.288 ± 0.096	1.504
HvUGP	0.168 ± 0.028	0.141 ± 0.041	1.187
AtUGP	0.539 ± 0.059	0.143 ± 0.027	3.754
StUGP	4.381 ± 1.231	0.297 ± 0.015	14.749
MeUGP	2.256 ± 0.252	0.042 ± 0.011	53.478
IbUGP	0.820 ± 0.094	0.130 ± 0.028	6.323
ZmUGP	0.811 ± 0.124	0.108 ± 0.028	7.479
DmUGP	0.0398 ± 0.005	0.097 ± 0.024	0.408
HsUGP	0.138 ± 0.027	0.208 ± 0.051	0.665

## Discussion

Among the 11 recombinant UGPases, most UGPases from plants showed higher specific activity and *k*_cat_/*K* values than those of UGPases from *E. coli*, budding yeast, *A. niger*, ScUGP, fruit fly, and human. This is an interesting phenomenon. The analysis of UGPase’s function must consider the enzyme’s product. UDP-Glc is an essential metabolite for the metabolism of carbohydrates in both photosynthetic and nonphotosynthetic tissues [42]. Among the six plant UGPases examined in this study, cassava MeUGP and potato StUGP are the two most active UGPases. Sweet potato IbUGP also exhibits high activity. They are high producers of starch-producing plants. Although ADP-glucose, synthesized by ADP-GPase, is the glucosyl donor for the elongation of alpha-1,4-glucosidic chains in plant starch synthesis, UGPases are closely related to starch accumulation. For example, with 70% less UGPase activity, the rice UGPase1 mutant demonstrated a reduction in starch content along with changes in starch properties of pasting and swelling [43], which may be related to the UGPase-derived Glc1P implicated in starch formation. Based on transcriptome sequencing data of storage root from sweet potato, sugar–starch conversion steps catalyzed by sucrose synthase and UGPase may be essential for starch accumulation [44].

In addition to its role in Suc pathways, UDP-Glc plays a crucial role, either directly or indirectly, in the biosynthesis of cell wall polysaccharides. The overexpression of the UGPase gene in jute (*Corchorus capsularis* L.) led to increased cellulose content compared with the control, while the lignin content remained unchanged [45]. Similarly, the overexpression of *Larix gmelinii* (dahurian Larch) UGPase gene resulted in enhanced vegetative growth in transgenic *Arabidopsis* and increased the contents of soluble sugars and cellulose and thickened parenchyma cell walls [46]. On the other hand, the *A. thaliana* UGPase1/UGPase2 double mutant displayed drastic growth defects, with absent callose deposition around microspores [19]. Thus, plants might need more active UGPases than microorganisms or animals to produce large quantities of UDP-Glc to support starch accumulation and the production of cell wall polysaccharides such as cellulose, hemicellulose, callose, and pectin.

Changes in the structure of UGPase have been suggested to play an essential role in regulating enzyme activity [20]. Despite structural similarities in the monomers of plant UGPase and other eukaryotic (human and yeast) UGPases, the oligomerization mold of plant UGPase with other eukaryotic UGPases is different. The monomer or mixture of monomer and dimer is the active form of plant UGPase [30,47], whereas octamers are an active form of human and yeast UGPases [12,24]. There may also be oligomerization differences between plant UGPases. *A. thaliana* UGPase forms a ‘head-to-toe’ dimer, where the N-terminal domain of one monomer faces the C-terminal domain of the other [30]. In contrast, sugarcane UGPase forms a ‘toe-to-toe’ dimer, where the C-terminal domain of the monomers interact with each other, similar to the arrangement of subunits in human and yeast UGPase [48]. Our study showed a high activity of MeUGP and StUGP. The StUGP protein structure remains still unknown, although there have been early reports of StUGP characterization [21,22]. Future research into the structures of StUGP and MeUGP may explain their high catalytic activity.

AnUGP was uncharacterized previously. The UGPases from filamentous fungi have yet to be extensively studied. Only a few reports addressed the transcriptional level of the UGPase gene and UGPase’s role in the mycelial development and virulence of pathogenic fungi [49,50]. The UGPase from model *Aspergillus nidulans* (AnidUGP) was not characterized until last year [51]. The specific activity of AnidUGP is low (0.41 U/mg). In contrast with other eukaryotic UGPases, AnidUGP displays a few structural variations [52]. The oligomerization mold of AnUGP compared with plant UGPases is different. AnidUGP displays intact octamers, which is also the typical active form of human and yeast UGPases [12,24]. In contrast, the monomer or mixture of monomers and dimer is an active form of plant UGPases [30,47]. Furthermore, despite AnidUGP exhibiting octamers as well as ScUGP and HsUGP, the AnidUGP cryoEM maps do not show proper four-fold symmetry [51]. In contrast, ScUGP and HsUGP show almost perfect four-fold symmetry in the crystal lattice [12,52]. There is a 96.4% similarity between AnUGP and AnidUGP. It is, therefore, speculated that AnUGP may share structural similarities with AnidUGP. Structural differences may account for AnUGP’s lower activity than other eukaryotic UGPases.

UDP-glucose, as the starting point for the synthesis of UDP-sugars (UDP-galactose and UDP-glucuronic acid), is a prerequisite for the rapid synthesis of any oligosaccharides containing these sugars, as well as other related compounds [53]. Thus, UGPases have been used as targets for metabolic engineering to improve the synthesis of products such as glycoglycerolipids, trehalose, hyperoside (quercetin 3-O-galactoside), rhodiola rosea, validatmycin A, and chondroitin-like capsular polysaccharide [54–59] or to produce specific products with unique properties like high molecular weight (>1 MDa) of hyaluronic acid [60,61]. As a target for metabolic engineering, *E. coli* UGPase was primarily utilized in the studies above [54–59]. Given that UGPases from cassava and potato can be correctly expressed in *E. coli* and are more active than EcUGP, the results provide the potential to use MeUGP or StUGP as the engineering target of cell factories instead of EcUGP.

Overall, recombinant UGPases were not thermally stable. Among 11 UGPases, ten UGPases were rapidly inactivated at 60°C, except for IbUGP. In particular, EcUGP and HsUGP showed a loss of activity after 2 h at 37°C. The results are unexpected, as 37°C is both the ideal growth temperature for *E. coli* and the typical body temperature for humans. It is still uncertain whether the buffer *in vitro* is unsuitable for maintaining UGPase activity. Our studies have shown that the optimal temperature for most recombinant UGPases is 37°C, which is consistent with findings for UGPases from other species such as *Streptococcus equi*, *Caenorhabditis elegans*, and *Danio rerio* [31,62]. The optimal temperature for most UGPases is known to be 21–37°C, which also suggests the instability of UGPases. It is known that a thermostable UGPase with the highest optimal temperature (80°C) is from thermophilic archaeon *Sulfolobus tokodaiibacteriafrom* [17]. The UGPase from soil actinobacterium *Thermocrispum agreste* was reported thermostable [63]. It is worthwhile to conduct further research to obtain highly active and stable UGPases to obtain UDP-glucose *in vitro*.

## Conclusions

Using the same assay method, the activities of 11 UGPases from bacteria, fungi, plants, and animals were compared. Plant UGPases commonly have higher activity than others. The top two with the highest enzyme activity are UGPases from potato and cassava, which also show high *k*_cat_/*K* value. They can potentially be used as the engineering targets of cell factories.

## Data Availability

The data underlying this article are available in the article and in its online supplementary material.

## Abbreviations

AnUGP: *Aspergillus niger* UGPase
AtUGP: *Arabidopsis thaliana* UGPase
DmUGP: *Drosophila melanogaster* UGPase
EcUGP: *Escherichia coli* UGPase
HsUGP: *Homo sapiens* UGPase
HvUGP: *Hordeum vulgare* UGPase
IbUGP: *Ipomoea batatas* UGPase
MeUGP: *Manihot esculenta* UGPase
PPi: pyrophosphate
ScUGP: *Saccharomyces cerevisiae* UGPase
StUGP: *Solanum tuberosum* UGPase
UGPases: UDP-glucose pyrophosphorylases
ZmUGP: *Zea mays* UGPase
